# Migratory Passerine Birds as Reservoirs of Lyme Borreliosis in Europe

**DOI:** 10.3201/eid1207.060127

**Published:** 2006-07

**Authors:** Pär Comstedt, Sven Bergström, Björn Olsen, Ulf Garpmo, Lisette Marjavaara, Hans Mejlon, Alan G. Barbour, Jonas Bunikis

**Affiliations:** * Umeå University, Umeå, Sweden;; † Kalmar University, Kalmar, Sweden;; ‡ Kalmar Hospital, Kalmar, Sweden;; § Uppsala University, Uppsala, Sweden;; ¶ University of California, Irvine, California, USA

**Keywords:** Spirochaetales, *Borrelia*, transmission, infection, competence, tick, infestation, Lyme disease, zoonosis, Sweden, research

## Abstract

To define the role of birds as reservoirs and disseminators of *Borrelia* spirochetes, we characterized tick infestation and reservoir competence of migratory passerine birds in Sweden. A total of 1,120 immature *Ixodes ricinus* ticks were removed from 13,260 birds and assayed by quantitative polymerase chain reaction (PCR) for *Borrelia*, followed by DNA sequencing for species and genotype identification. Distributions of ticks on birds were aggregated, presumably because of varying encounters with ticks along migratory routes. Lyme borreliosis spirochetes were detected in 160 (14%) ticks. *Borrelia garinii* was the most common species in PCR-positive samples and included genotypes associated with human infections. Infestation prevalence with infected ticks was 5 times greater among ground-foraging birds than other bird species, but the 2 groups were equally competent in transmitting *Borrelia*. Migratory passerine birds host epidemiologically important vector ticks and *Borrelia* species and vary in effectiveness as reservoirs on the basis of their feeding behavior.

## Abstract 2

Birds host vector ticks and *Borrelia* species and vary in effectiveness as reservoirs.

Recent outbreaks of West Nile virus infection or avian influenza indicate that birds participate in the ecology of zoonotic infections, an important cause of illness and death in humans and animals (*1*). The emergence of these threats underscores the need for understanding the maintenance of bird-associated infections in nature, which is prerequisite for disease prevention.

Migratory birds are known to carry several microbial agents of human disease, including viruses, chlamydiae, and enterobacteria (*2**,**3*). Evidence of the last 2 decades indicates that birds in North America and Eurasia host vectorborne pathogens, such as *Anaplasma* species and Lyme borreliosis (LB) spirochetes (*4**–**6*). LB is the most common vectorborne zoonosis in temperate regions of the Northern Hemisphere and is transmitted to humans by *Ixodes* ticks (*7*). *Borrelia* spirochetes infect naive *Ixodes* larvae when they feed on a reservoir host and are transmitted back to the reservoir population by infected nymphs. Rodent species, such as the white-footed mouse (*Peromyscus leucopus*) in the northeastern United States and *Apodemus* and *Clethrionomys* species in continental Europe, are common hosts of both immature ticks and LB spirochetes (*8**,**9*). However, recent field vaccination and biodiversity studies suggest that alternative hosts play a greater role than expected in the natural cycle of LB (*10**,**11*).

In comparison with studies of mammals as LB reservoirs, few studies have been conducted on the role of birds as hosts of *Borrelia*. The natural cycle of LB spirochetes, in particular *Borrelia garinii*, involves seabirds in northern Europe and game birds in the United Kingdom, which are the most studied models (*12**,**13*). However, the relationship between migratory passerine birds and *Borrelia* is less understood. Although experimental studies on avian infection have been conducted (*14**–**17*), less is known about reservoir competence of natural bird populations, especially those that could transmit ticks that frequently bite humans (*5**,**18**–**20*).

Information that would allow comparison of the reservoir importance of bird and other vertebrate populations is not available or is controversial. Although 1 modeling study found that the frequency of LB cases was positively correlated with species diversity of ground-dwelling birds (*21*), other studies have found the contribution of birds in hosting and infecting ticks to be low (*22**,**23*). Another uncertainty is epidemiologic implications of LB group spirochetes associated with birds. For example, birds in Europe are reservoirs of *B*. *valaisiana*, which has not been associated with disease (*19*).

In the present study, we characterized tick infestation and *Borrelia* transmission from migratory passerine birds captured in southern Sweden to further define their importance as reservoirs and disseminators of these spirochetes. We found that these birds are hosts of epidemiologically important vector ticks and *Borrelia* species. However, exposure of birds to ticks, which depends on feeding habits, determines their effectiveness as *Borrelia* reservoirs.

## Materials and Methods

### Bird Capture and Tick Collection

**Figure 1:**
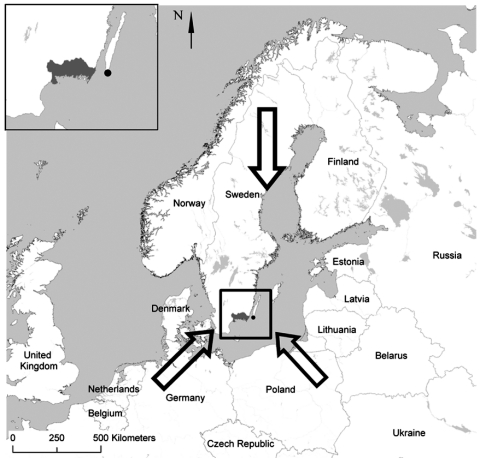
Scandinavian Peninsula in northern Europe. Location of Ottenby Bird Observatory (solid circle) on the southern tip of Öland Island in the Baltic Sea and nearby Blekinge County (shaded area) in mainland southern Sweden are shown in the inset. Directions of bird migration northward from outside northern Europe in the spring and back from Scandinavia and western Russia in the fall are shown by large arrows.

Birds were captured at Ottenby Bird Observatory (http://www.sofnet.org/ofstn/Engelska) at the southern point of Öland Island in the Baltic Sea (56°12´N, 16°24´E) southeast of the Swedish mainland (Figure 1). Japanese mist nets and Helgoland traps were used for capture as previously described (*5*), and with the approval of the Swedish Museum of Natural History, Stockholm. Birds were trapped from March 17 to May 30, and from July 7 to November 13 of 2001, periods that are representative of spring and fall migrations, respectively. Trapped birds were banded and examined daily for ticks during these periods, except on April 2, September 17, 18, 22, and 24, and November 14, 16, and 17. Recaptured birds were not studied. Ticks attached to a bird's head were removed and, after species and stage identification, stored individually at -70°C.

### DNA Extraction and Quantitative Real-Time PCR

Tick DNA was extracted by using the Puregene DNA isolation protocol (Gentra Systems, Minneapolis, MN, USA) and stored at -20°C. DNA extracts were assayed for LB and relapsing fever (RF) group *Borrelia* by using a quantitative real-time polymerase chain reaction (qPCR) assay with probes and primers specific for the 16S rRNA gene (*11*). Serially diluted *B*. *burgdorferi* B31 and *B*. *hermsii* HS1 DNA were used as standards (*11*).

### Identifying and Genotyping *Borrelia* Species

*Borrelia* species were identified by direct sequencing of the amplicons generated from the *rrs* (16S)-*rrl* (23S) intergenic spacer (IGS) or 16S gene PCRs (*24**,**25*). When necessary, nested modification of these assays was used to increase success of amplification. In addition, we obtained *rrs*-*rrl* IGS sequences of *B*. *garinii* isolated from skin biopsy specimens of erythema migrans lesions from 11 LB patients from southern Sweden (*26*). Positions with at least 2 different character states in >2 sequences each were considered polymorphic and included in the typing matrix. Sequences of new *B*. *garinii* IGS variants were deposited in GenBank database under accession nos. DQ307372–DQ307377.

### Statistical Analyses

We used simple regression analysis, nonparametric Mann-Whitney U test, and standard parametric statistics giving the mean ± 95% confidence intervals (CIs) for continuous variables. We also used Fisher exact test, χ^2^ goodness of fit, and odds ratio (OR) procedures for proportions. Statistical analyses were conducted with StatView version 5.0.1 (SAS Institute Inc., Cary, NC, USA) and StatXact version 6 (Cytel Software, Cambridge, MA, USA).

## Results

### Tick Infestation of Birds

According to the Ornithological Council's list of avian orders (available at http://www.nmnh.si.edu/BIRDNET/ORDERS/), 13,123 birds captured in this study were passerines (Passeriformes) (Table 1). In addition, there were 83 great spotted woodpeckers (Piciformes) and 54 sparrowhawks (Falconiformes). All studied birds were migratory. The 38 bird species studied comprised 6 ecologic guilds (*27*), each defined by a bird's foraging behavior. Three guilds comprised 19 species of ground-foraging birds and included 4,614 invertebrate feeders, 906 granivores, and 125 insectivores. In addition, 500 wrens and 30 marsh warblers, which are herbaceous plant–foraging insectivores that predominantly feed on the ground, were included in this group. The remaining 3 guilds and 17 species, referred to as other birds, comprised 223 raptors, 6,612 arboreal insectivores, and 250 other reed-foraging insectivores.

**Table 1:** Infestation of migratory birds *by Ixodes ricinus* ticks and tick infection with Lyme borreliosis group spirochetes, Ottenby Bird Observatory study, Sweden, 2001

Bird species*		No. birds	No. ticks	No. (%) birds infested	Mean no. ticks/ infested bird	No. (%) birds with infected ticks	No. larvae	No. (%) positive larvae	No. nymphs	No. (%) positive nymphs
Ground foraging										
	*Erithacus rubecula*	3,939	446	185 (5)	2.4	20 (11)	296	6 (2)	150	15 (10)
	*Luscina luscinia*	32	9	4 (13)	2.3	2 (50)	5	0	4	2 (50)
	*Luscina svecica*	85	8	5 (6)	1.6	1 (20)	0	0	8	1 (13)
	*Turdus philomelus*	261	141	24 (9)	5.9	10 (42)	88	14 (16)	53	17 (32)
	*Turdus iliacus*	51	22	9 (18)	2.4	2 (22)	5	0	17	4 (24)
	*Turdus merula*	193	170	44 (23)	3.9	15 (34)	36	11 (31)	134	28 (21)
	*Turdus pilaris*	23	6	3 (13)	2	1 (33)	3	1 (33)	3	1 (33)
	*Sturnus vulgaris*	30	18	9 (30)	2	2 (22)	7	3 (43)	11	4 (36)
	*Prunella modularis*	64	9	4 (6)	2.3	1 (25)	2	0	7	1 (14)
	*Anthus trivialis*	61	29	11 (18)	2.6	6 (55)	17	8 (47)	12	6 (50)
	*Aluada arvensis*	1	6	1 (100)	6	1 (100)	6	1 (17)	0	0
	*Fringilla coelebs*	122	9	2 (2)	4.5	1 (50)	8	8 (100)	1	0
	*Carduelis flammea*	441	1	1 (0.2)	1	0	1	0	0	0
	*Carduelis spinus*	79	1	1 (1)	1	0	0	0	1	0
	*Pyrrhula pyrrhula*	55	8	5 (9)	1.6	2 (40)	1	0	7	2 (29)
	*Carduelis chloris*	73	5	5 (7)	1	0	1	0	4	0
	*Carduelis cannabina*	26	1	1 (4)	1	0	0	0	1	0
	*Carpodacus erythrinus*	55	1	1 (2)	1	0	0	0	1	0
	*Emberiza schoeniclus*	54	1	1 (2)	1	0	1	0	0	0
	*Troglodytes troglodytes*	500	33	17 (3)	1.9	0	25	0	8	0
	*Acrocephalus palustris*	30	1	1 (3)	1	0	0	0	1	0
Other										
	*Accipiter nisus*	54	2	1 (2)	2	0	0	0	2	0
	*Lanius collurio*	169	7	2 (1)	3.5	0	4	0	3	0
	*Dendrocopus major*	83	8	1 (1)	8	1 (100)	2	0	6	4 (67)
	*Hippolais icterina*	87	15	2 (2)	7.5	0	15	0	0	0
	*Sylvia atricapilla*	170	8	7 (4)	1.1	0	4	0	4	0
	*Sylvia borin*	194	1	1 (0.5)	1	0	0	0	1	0
	*Sylvia curruca*	621	11	8 (1)	1.4	2 (25)	4	0	7	2 (29)
	*Sylvia nisoria*	13	4	3 (23)	1.3	0	1	0	3	0
	*Phylloscopus sibilatrix*	65	1	1 (2)	1	0	1	0	0	0
	*Phylloscopus trochilus*	2,116	21	19 (1)	1.1	1 (5)	9	0	12	1 (8)
	*Regulus regulus*	2,212	1	1 (0.1)	1	0	0	0	1	0
	*Parus major*	132	35	19 (14)	1.8	9 (47)	22	6 (27)	13	5 (39)
	*Parus caeruleus*	541	9	6 (1)	1.5	0	1	0	8	0
	*Certhia familiaris*	37	1	1 (3)	1	0	0	0	1	0
	*Phoenicurus phoenicurus*	341	23	12 (4)	1.9	2 (17)	12	0	11	2 (18)
	*Sylvia communis*	220	47	18 (8)	2.6	3 (17)	29	3 (10)	18	4 (22)
	*Acrocephalus scirpaceus*	30	1	1 (3)	1	0	0	0	1	0
Total		13,260	1,120	437 (3)	2.6	82 (19)	606	61 (10)	514	99 (19)

*Ground-foraging species include invertebrate feeders (*Erithacus rubecula* through *Sturnus vulgaris*), insectivores (*Prunella modularis* and *Anthus trivialis*), granivores (*Aluada arvensis* through *Emberiza schoeniclus*), and herbaceous plant–foraging insectivores (*Troglodytes troglodytes* and *Acrocephalus palustris*). Other species include raptors (*Accipiter nisus* and *Lanius collurio*), arboreal insectivores (*Dendrocopus major* through *Phoenicurus phoenicurus*), and reed-foraging insectivores (*Sylvia communis* and *Acrocephalus scirpaceus*). The common names of the 38 bird species listed (from top to bottom) are European robin, thrush nightingale, bluethroat, song thrush, redwing thrush, blackbird, fieldfare, starling, dunnock, tree pipit, skylark, chaffinch, redpoll, siskin, bull finch, green finch, linnet, scarlet rosefinch, reed bunting, wren, marsh warbler, sparrow hawk, red-backed shrike, great spotted woodpecker, icterine warbler, blackcap, garden warbler, lesser whitethroat, barred warbler, wood warbler, willow warbler, goldcrest, great tit, blue tit, tree creeper, redstart, whitethroat, and reed warbler.

**Figure 2:**
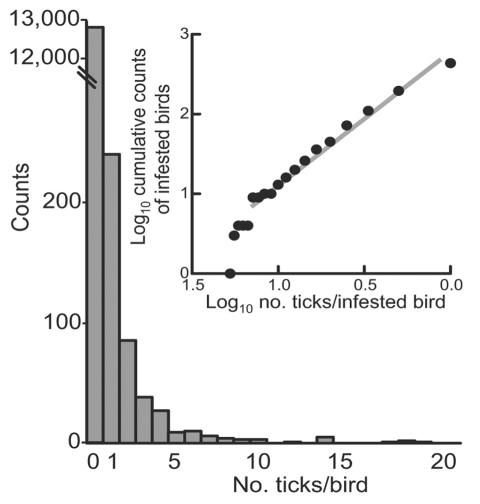
Frequency distribution of subadult tick infestations of migratory birds captured at Ottenby Bird Observatory, Sweden, 2001. Aggregation of risk of infestation is shown in the inset. Three birds with 26, 33, or 40 ticks, most of which were larvae, are excluded from the figure.

We measured bird infestation with ticks and then compared the occurrence of the ticks on the birds with different foraging habits. Overall, 1,127 ticks were removed from 437 (3.3%) of 13,260 birds (Table 1). Of these ticks, 606 (54%) were larvae, 514 (46%) were nymphs, and 7 (0.6%) were adults of *Ixodes ricinus*, confirming that subadult ticks predominate on birds (*5*). (Because of their low number, the adult ticks, as well as 4 *I*. *lividus* nymphs removed from 1 bird, were excluded from further analyses.). *I*. *ricinus* larvae and nymphs were found on 226 (52%) and 310 (71%) of 437 infested birds, respectively; 99 (23%) of these birds were infested with both stages. The proportion of birds infested with larvae was higher in fall than in spring: 188 (2.1%) of 9,145 birds versus 38 (0.9%) of 4,115 birds (OR 2.3, 95% CI 1.6–3.2). In contrast, the proportion of birds infested with nymphs was similar between the 2 collection periods: 212 (2.3%) birds in fall and 98 (2.4%) in spring (OR 1.0, CI 0.8–1.2). The counts of captured birds with no ticks or >1 subadult tick followed a negative binomial distribution and are shown in Figure 2. The counts of these ticks on infested birds more specifically corresponded to a Zipf distribution (Kolmogorov-Smirnov statistic 0.05, p = 0.3; inset in Figure 2). Aggregation of infestation risk was further indicated by the finding that once a bird is infested with 1 subadult tick, the likelihood of infestation with >2 such ticks was higher than expected from a Poisson distribution (p<0.0001).

Among infested birds, no correlation was found (*R* = 0.01) between the number of larvae and nymphs on a given bird, which is an indication that most larvae and nymphs were not host-seeking at the same time and place. Further support for this conclusion was an observed count of 99 birds co-infested with nymphs and larvae that was 38% lower than expected, if larval and nymphal infestations were fully covariant (*z* = 4.96, p<0.001). Co-infestation was lower than expected among both spring and fall migrants, especially in the latter group (*z* = 4.44, p<0.001). With regard to risk for infestation among different types of birds, prevalence was greater among ground foragers than other birds by group (335 [5.4%] of 6,175 vs. 102 [1.4%] of 7,085, OR 3.9; 95% CI 3.1–4.9). Infestation also differed by individual species (p<0.02, by Mann-Whitney U test) (Table 1).

We then retrospectively analyzed data on infestations of 15,839 birds captured in Scandinavia in 1991 that matched the species composition of this study (Table 2) (*5*). Similar to findings in the present collection, infestations with subadult stages were ≈3-fold more common among ground foragers (297 [3.5%] of 8,388) than in other birds by group (100 [1.3%] of 7,451, OR 2.7, 95% CI 2.1–3.4) and by individual species (p<0.02, by Mann-Whitney U test).

**Table 2:** *Ixodes ricinus* ticks on migratory birds at bird observatories in Sweden and Denmark, 1991

Bird species*	No. birds examined	No. ticks collected	No. (%) birds infested	Mean no. ticks per infested bird
Ground foraging				
	*Erithacus rubecula*	3,345	145	72 (2.2)	2.0
	*Luscina luscinia*	204	90	28 (13.7)	3.2
	*Luscina svecica*	301	18	11 (3.7)	1.6
	*Turdus philomelus*	610	50	22 (3.6)	2.3
	*Turdus iliacus*	457	100	38 (8.3)	2.6
	*Turdus merula*	264	121	44 (16.7)	2.8
	*Turdus pilaris*	109	5	4 (3.7)	1.3
	*Sturnus vulgaris*	18	9	5 (27.7)	1.8
	*Prunella modularis*	68	7	4 (5.8)	1.8
	*Anthus trivialis*	237	105	47 (19.8)	2.2
	*Fringilla coelebs*	169	3	3 (1.7)	1.0
	*Carduelis flammea*	1,300	5	2 (0.2)	2.5
	*Pyrrhula pyrrhula*	196	4	2 (1.0)	2.0
	*Carduelis chloris*	93	1	1 (1.1)	1.0
	*Emberiza schoeniclus*	187	2	2 (1.1)	1.0
	*Troglodytes troglodytes*	674	12	6 (0.9)	2.0
	*Acrocephalus palustris*	156	8	6 (3.8)	1.3
Other				
	*Accipiter nisus*	93	2	2 (2.2)	1.0
	*Sylvia atricapilla*	501	11	5 (1.0)	2.2
	*Sylvia borin*	285	1	1 (0.3)	1.0
	*Sylvia curruca*	450	4	4 (0.9)	1.0
	*Regulus regulus*	2,566	5	5 (0.2)	1.0
	*Parus major*	454	2	2 (0.4)	1.0
	*Phoenicurus phoenicurus*	688	70	44 (6.4)	1.6
	*Sylvia communis*	650	38	22 (3.4)	1.7
	*Acrocephalus scirpaceus*	1,764	15	15 (0.9)	1.0
Total	15,839	833	397 (2.5)	2.1

*Ground-foraging species include invertebrate feeders (*Erithacus rubecula* through *Sturnus vulgaris*), insectivores (*Prunella modularis* and *Anthus trivialis*), granivores (*Fringilla coelebs* through *Emberiza schoeniclus*), and herbaceous plant–foraging insectivores (*Troglodytes troglodytes* and *Acrocephalus palustris*). Other species include raptors (*Accipiter nisus*), arboreal insectivores (*Sylvia atricapilla* through *Phoenicurus phoenicurus*), and reed-foraging insectivores (*Sylvia communis* and *Acrocephalus scirpaceus*). The common names of the 26 species listed (from top to bottom) are European robin, thrush nightingale, bluethroat, song thrush, redwing thrush, blackbird, fieldfare, starling, dunnock, tree pipit, chaffinch, redpoll, bull finch, green finch, reed bunting, wren, marsh warbler, sparrow hawk, blackcap, garden warbler, lesser whitethroat, goldcrest, great tit, redstart, whitethroat, and reed warbler.

### *Borrelia* Infection of Ticks

To characterize the role of birds in transmitting spirochetes to ticks, we determined the prevalence of *Borrelia* infection in larvae and nymphs by using multiplex qPCR, which also differentiates between LB and RF group spirochetes. LB spirochetes were found in 160 (14%) of 1,120 subadult *I*. *ricinus*, and were more common among nymphs than larvae (19.3% vs. 10.1%, OR 2.1, 95% CI 1.5–3.1) (Table 1), presumably a result of accumulation of infection in the former stage during consecutive feedings. Three samples (0.3%), 1 larva and 2 nymphs, were positive by qPCR for RF organisms.

LB spirochetes are rarely transmitted transovarially or during co-feeding (*28**,**29*), and their detection in feeding larvae is presumptive evidence of acquisition from the larva's host. To demonstrate that larvae in this study were infected by the birds, we analyzed 226 larvae-infested birds by comparing the proportion of the birds with infected larvae among the birds with a single larva (singly infested) and the proportion of the birds with >1 infected larva among the birds infested with >2 larvae (multiply infested). If the spirochetes were acquired transovarially, these indicators would not be expected to differ between the 2 groups. Conversely, a higher prevalence of infection in ticks from multiply infested birds in comparison to ticks from singly infested birds would be evidence of transmission from birds. Consistent with the latter hypothetical outcome, the proportions in singly infested and multiply infested birds were 7 (5.5%) of 128 and 21 (21.4%) of 98, respectively (OR 4.7, 95% CI 1.9–11.6).

In another approach with multiply infested birds, we compared the count of infected larvae expected at 5.5% prevalence of infection (as found for the larvae of singly infested birds) with that observed in the larvae after the first positive larva has been identified. The observed and expected count of positive larvae was 33 and 6, respectively (p = 0.004), which is additional evidence of transmission of spirochetes from birds to larvae.

**Figure 3:**
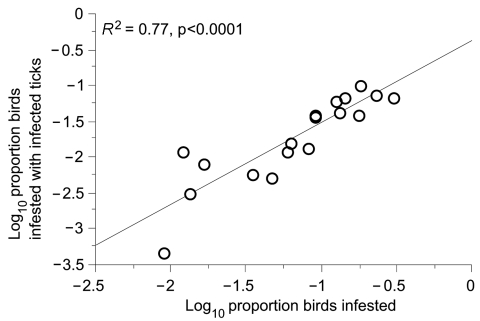
Relationship between tick infestation of birds and infestation with ticks infected with Lyme borreliosis group spirochetes.

Excluding the 1 skylark in the study, infestation by infected ticks was higher (3.0%) in 20 ground-foraging species than in 17 other species (0.6%) (p<0.05, by t-test) (Table 1). These rates correlated with the overall infestation rate of birds (*R*^2^ = 0.77; p<0.001), which is another indication that ticks were being infected by birds (Figure 3). We also compared the frequency of infection among birds in the 2 groups by measuring the ratio of birds with infected larvae to the number of larvae-infested birds. Infection with LB spirochetes was more common in ground-foraging birds than in other bird species: 17 (34.0%) of 50 birds of 12 species versus 5 (7.8%) of 64 birds of 13 species, respectively (p<0.001, OR 6.1, 95% CI 2.1–18.0). We excluded from this analysis 106 European robins (*Erithacus rubecula*), which predominated among ground foragers but were infested by larvae with an unusually low infection prevalence of 2%.

### *Borrelia* Species Composition

Eighty-eight (55%) of 160 samples that were positive by qPCR with LB probe produced amplicons in *rrs*-*rrl* IGS or 16S PCR. The latter PCR was performed on 12 samples that in qPCR with LB group-specific probe showed a distinct amplification pattern presumably attributable to *B*. *valaisiana* DNA and were negative in the IGS PCR. Sequence analysis of the amplicons showed *B*. *garinii* in 75 (85%) samples, *B*. *valaisiana* in 6 (7%) samples, *B*. *afzelli* in 4 (5%) samples, and *B*. *burgdorferi* in 3 (3%) samples (Table 3). The 3 samples positive by qPCR with the probe for RF spirochetes were identified by *rrs*-*rrl* IGS sequencing as *B*. *miyamotoi* group spirochetes (*30*).

**Table 3:** *Borrelia* species in *Ixodes ricinus* ticks from migratory birds

			Larvae	Nymphs	Total
No. ticks tested*			606	514	1,120
No. (%) positive					
	LB group†		61 (10.1)	99 (19.3)	160 (14.3)
		*B. garinii*	27	48	75
		*B. burgdorferi*	1	2	3
		*B. afzelli*	0	4	4
		*B. valaisiana*	1	5	6
	RF group†		1 (0.2)	2 (0.4)	3 (0.3)
		*B. miyamotoi*	1	2	3

*Quantitative polymerase chain reaction for Lyme borreliosis (LB) and relapsing fever (RF) including *B. miyamotoi* group spirochetes (*11*). †*Borrelia* species was determined for 88 of 160 LB-positive samples and all 3 RF-positive samples by sequencing a partial rrs-rrl intergenic spacer region region (*25*) or, for *B. valaisiana*, a partial 16S rRNA gene (*24*).

To determine epidemiologic importance of *B*. *garinii* variants that are disseminated or maintained by migratory birds, we typed and compared the *rrs*-*rrl* IGS region of 47 of 75 *B. garinii* samples from bird ticks and 11 erythema migrans isolates of this species from LB patients from nearby Blekinge County in mainland Sweden (Figure 1). *B*. *garinii* PCR samples from ticks produced 11 variants; 6 of these variants, represented by 31 (66%) samples, were also found in LB patients. Larvae were infected with 3 *B*. *garinii* variants also found in biopsy specimens, which indicates that migratory birds serve as hosts for *B*. *garinii* strains that are pathogenic to humans.

### Reservoir Competence of Migratory Birds

**Figure 4:**
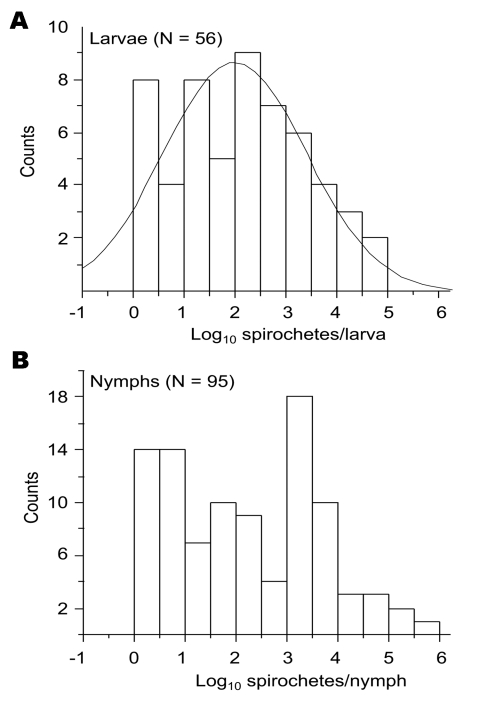
Frequency distribution of Lyme borreliosis group spirochete load in larvae (A) and nymphs (B). Normal comparison for the distribution of spirochete counts in larvae is shown. Values <1 cell/tick found in 5 larvae and 4 nymphs are excluded from the analysis.

With the exception of pheasants in the United Kingdom (*13*), the reservoir competence of other bird groups or species, including migratory birds, is not fully understood (*31*). The efficiency of transmission of spirochetes, as measured by their prevalence in ticks, is 1 correlate of vertebrate host competence in maintaining the natural cycle of LB (*17*). To assess such competence of migratory birds, we measured and compared the spirochete count and infection prevalence in larvae and nymphs collected from these birds. Inasmuch as birds migrate in regularly alternating periods of 1 day resting and 6 days flying (*32*), we presumed that the ticks collected from the birds represent a random collection with respect to the degree of their engorgement. The frequency of spirochete counts in the larvae followed a normal distribution (Figure 4). In contrast, it was bimodal for the nymphs, which suggests that 2 populations of this stage are present: 1 with low spirochete counts and 1 with higher spirochete counts.

**Figure 5:**
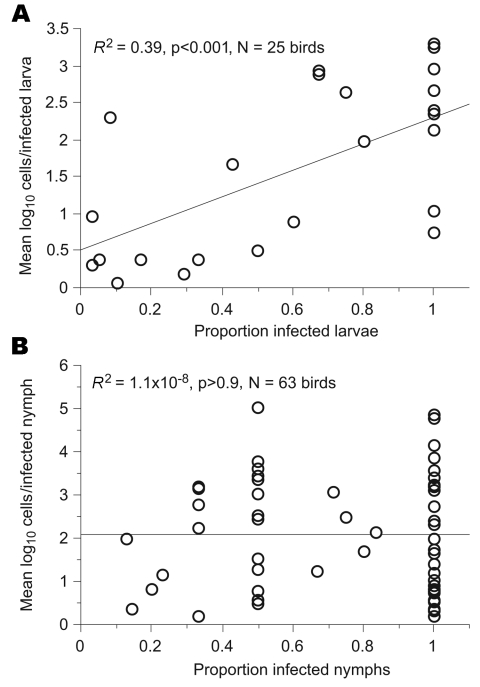
Relationship between Lyme borreliosis spirochete load and proportion of infected larvae (A) and nymphs (B). Values <1 cell/tick were excluded from the analysis.

To further distinguish between infections of larvae and nymphs, we compared the 2 stages with respect to the correlation between the spirochete load and infection prevalence among ticks collected from the same bird. These 2 variables showed a correlation for 56 larvae from 25 birds (*R*^2^ = 0.39, p<0.01) but not for 95 nymphs collected from 63 birds (*R*^2^<0.01, p>0.5) (Figure 5).

We next evaluated ground-foraging birds and birds of other species for efficiency of spirochete transmission to larvae by comparing the infection prevalence of larvae from individual birds. Twenty-three birds of 8 ground-foraging species and 5 birds of 2 other species were available for this analysis. The mean infection prevalence of individual collections of larvae from ground foragers and other birds was 61% (95% CI 46%–77%) and 77% (50%–100%), respectively, (p>0.4). To validate this result, which suggests that migratory passerines transmit LB spirochetes to ticks with similar efficiency, we compared LB spirochete counts in the larvae from the 2 bird groups. The cell counts were available for 52 larvae from 25 ground foragers and 9 larvae from 5 birds of other species. Weighted means of spirochetes per infected larvae from ground-foraging birds and other bird species were 135 (95% CI 21–862) and 23 (95% CI 2–318), respectively (p = 0.4). This was additional evidence that the 2 bird groups were equally competent in transmitting infection to larvae.

## Discussion

This was the first large-scale study to show that migratory passerine birds participate in the enzootic maintenance of *Borrelia* spirochetes, including species and genotypes associated with LB in humans. By combining 2 approaches, quantification of infection in vector ticks and molecular typing, we demonstrate that these birds constitute an epidemiologically important alternative reservoir of LB, as well as a means for wide distribution of the pathogen.

This study's approach of characterizing *Borrelia* infection of ticks engorged on birds is analogous to xenodiagnosis, which is commonly used in assessing reservoir competence in the laboratory (*17*). A correlation between rate of tick infestation and infestation with infected ticks is evidence of a bird source of infection. Consistent with this source, the proportion of birds infested with multiply infected larvae and the observed counts of infected larvae on individual birds exceeded the baseline values assumed to represent a hypothetical transovarial transmission. Furthermore, infection prevalence correlated with the number of spirochetes in larvae, which suggests a new variable for quantifying reservoir competence for *Borrelia* transmission. Finally, *Borrelia* species composition in larvae, namely, predominance of *B*. *garinii* and absence of *B*. *afzelii*, indicates the bird source of infection (*33*). However, inferring reservoir competence from measuring infection of naturally infesting ticks has drawbacks. Collection of only birds that had ticks on them at the time of capture could lead to an underestimation of the prevalence of infection among the studied bird population. Also, in this study we could not follow-up and quantify the infection of the nymphs that emerge from infected larvae, a transition that determines the ability of the nymphs to infect other hosts during subsequent feeding (*17*).

A negative binomial distribution of natural loads of subadult *I*. *ricinus* on migratory birds is a common characteristic of ectoparasitism (*34**,**35*), including infestation with ticks (*36*). Similar to other hosts, infestation of migratory birds is nonrandom, presumably due to different tick densities at stopover sites along the migration routes. These routes likely run in a south–north direction and within boundaries of central and northeastern Europe. Two indications of this are infestation of birds almost exclusively with *I*. *ricinus* ticks, which prevail in these regions, and the absence of *I*. *persulcatus*, a common bird parasite in eastern Europe and Asia (*37*).

Different activation times of larvae and nymphs along this geoclimatic axis also determine the dissociation between infestations with the 2 stages, as indicated by lack of correlation between their numbers on a given bird, as well as relatively infrequent co-infestations. This dissociation is further supported by the evidence of distinct histories of infection with LB spirochetes of larvae and nymphs: 1) greater prevalence of infection in nymphs than in larvae; 2) correlation between prevalence of infection and spirochete counts in larvae, but not nymphs; and 3) bimodal distribution of spirochete counts in nymphs, but not larvae, presumably due to residual infection in the nymphs acquired during feeding at larval stage. Thus, the 2 subadult tick stages represent different aspects of migratory birds' involvement in the maintenance of *Borrelia*. Whereas both stages contribute to the assessment of geographic dissemination and carrying capacity of infected vector ticks by birds, larvae provide a direct measure of birds' competence in transmitting the spirochetes.

In comparison with other hosts, birds appear to be infested with fewer ticks (*19**,**22**,**38*). For example, the 2.1–2.6 ticks per infested bird density found in this study is ≈20–30 times less than that found on rodents in south-central Sweden (*39*). Conversely, migratory bird population estimates suggest that their actual contribution in hosting, infecting, and disseminating ticks may be at least as important as that of other hosts. For example, ≈150 million migratory passerine birds come to their breeding grounds in Sweden in the spring (*40*), and at least 2 times that number migrate in the fall. Assuming that our findings are representative of these bird populations and at observed infestation and infection rates, ≈15 million infested birds would disseminate 40 million ticks, of which 5.6 million would be infected with LB group spirochetes. Five million of these ticks would carry *B*. *garinii*, and at least one third would be infected by birds. The 16% extrapolated prevalence of *B*. *garinii* found in nymphs feeding on migratory passerines in this study corresponds to ≈50% of that found in pheasants in the United Kingdom, where these birds are the major reservoir of this spirochete (*13*). Thus, migratory passerines contribute to influx of *B*. *garinii* into the natural circulation, where this species is known to adapt to local enzootic transmission cycle involving mammals.

Measuring the occurrence of ticks in 2 uniquely large migratory bird collections in Scandinavia at a 10-year interval provided consistent evidence of greater risk for exposure to ticks among ground-foraging birds. As a result of this increased risk, the infestation rate with infected ticks and the proportion of presumably infected birds were greater in ground feeders than in other birds. However, the transmission of spirochetes from bird to tick, defined as the amount and prevalence of infection in ticks, was similar between the 2 migratory bird groups. Thus, a bird's feeding behavior, rather than other biologic differences, is a critical determinant of its reservoir potential. Notwithstanding exceptions and as a group, those birds that spend time on the ground contribute most effectively to the maintenance of both the vector ticks and the spirochetes.

The agent of LB in North America, *B*. *burgdorferi*, is associated with different vertebrate reservoirs, including birds (*4**,**31*). The American robin, an abundant and commonly tick-infested passerine, is as effective as mice in reservoir competence for this bacterium (*17*). Understanding the contribution of this and other alternative reservoirs in enzootic maintenance of *B*. *burgdorferi* is prerequisite for advancing prevention strategies for LB (*11*).

## References

[1] Woolhouse MEJ, Gowtage-Sequeria S. Host range and emerging and reemerging pathogens. Emerg Infect Dis. 2005;11:1842–7. 16485468 10.3201/eid1112.050997 PMC3367654

[2] Palmgren H, Sellin M, Bergström S, Olsen B. Enteropathogenic bacteria in migrating birds arriving in Sweden. Scand J Infect Dis. 1997;29:565–8. 10.3109/00365549709035895 9571735

[3] Olsen B, Persson K, Broholm KA. PCR detection of *Chlamydia psittaci* in faecal samples from passerine birds in Sweden. Epidemiol Infect. 1998;121:481–4. 10.1017/S0950268898001320 9825803 PMC2809549

[4] Anderson JF, Johnson RC, Magnarelli LA, Hyde FW. Involvement of birds in the epidemiology of the Lyme disease agent *Borrelia burgdorferi.* Infect Immun. 1986;51:394–6. 3943893 10.1128/iai.51.2.394-396.1986 PMC262337

[5] Olsen B, Jaenson TG, Bergström S. Prevalence of *Borrelia burgdorferi* sensu lato-infected ticks on migrating birds. Appl Environ Microbiol. 1995;61:3082–7. 7487041 10.1128/aem.61.8.3082-3087.1995 PMC167585

[6] Bjoersdorff A, Bergström S, Massung RF, Haemig PD, Olsen B. *Ehrlichia*-infected ticks on migrating birds. Emerg Infect Dis. 2001;7:877–9. 10.3201/eid0705.010517 11747702 PMC2631880

[7] CDC. Lyme disease—United States, 1996. MMWR Morb Mortal Wkly Rep. 1997;46:531–5. 9191035

[8] Levine JF, Wilson ML, Spielman A. Mice as reservoirs of the Lyme disease spirochete. Am J Trop Med Hyg. 1985;34:355–60. 3985277 10.4269/ajtmh.1985.34.355

[9] Humair PF, Rais O, Gern L. Transmission of *Borrelia afzelii* from *Apodemus* mice and *Clethrionomys* voles to *Ixodes ricinus* ticks: differential transmission pattern and overwintering maintenance. Parasitology. 1999;118:33–42. 10.1017/S0031182098003564 10070659

[10] Ostfeld RS, Keesing F. Biodiversity and disease risk: the case of Lyme disease. Conserv Biol. 2000;14:722–8. 10.1046/j.1523-1739.2000.99014.x

[11] Tsao JI, Wootton JT, Bunikis J, Luna MG, Fish D, Barbour AG. An ecological approach to preventing human infection: vaccinating wild mouse reservoirs intervenes in the Lyme disease cycle. Proc Natl Acad Sci U S A. 2004;101:18159–64. 10.1073/pnas.0405763102 15608069 PMC536054

[12] Olsen B, Jaenson TG, Noppa L, Bunikis J, Bergström S. A Lyme borreliosis cycle in seabirds and *Ixodes uriae* ticks. Nature. 1993;362:340–2. 10.1038/362340a0 8455718

[13] Kurtenbach K, Peacey M, Rijpkema SG, Hoodless AN, Nuttall PA, Randolph SE. Differential transmission of the genospecies of *Borrelia burgdorferi* sensu lato by game birds and small rodents in England. Appl Environ Microbiol. 1998;64:1169–74. 9546150 10.1128/aem.64.4.1169-1174.1998 PMC106125

[14] Olsen B, Gylfe A, Bergström S. Canary finches (*Serinus canaria*) as an avian infection model for Lyme borreliosis. Microb Pathog. 1996;20:319–24. 10.1006/mpat.1996.0030 8831827

[15] Humair PF, Postic D, Wallich R, Gern L. An avian reservoir (*Turdus merula*) of the Lyme borreliosis spirochetes. Zentralbl Bakteriol. 1998;287:521–38. 9638881

[16] Gylfe A, Bergstrom S, Lundström J, Olsen B. Reactivation of *Borrelia* infection in birds. Nature. 2000;403:724–5. 10.1038/35001663 10693792

[17] Richter D, Spielman A, Komar N, Matuschka FR. Competence of American robins as reservoir hosts for Lyme disease spirochetes. Emerg Infect Dis. 2000;6:133–8. 10.3201/eid0602.000205 10756146 PMC2640847

[18] Humair PF, Turrian N, Aeschlimann A, Gern L. *Ixodes ricinus* immatures on birds in a focus of Lyme borreliosis. Folia Parasitol (Praha). 1993;40:237–42. 8314179

[19] Hanincova K, Taragelova V, Koci J, Schafer SM, Hails R, Ukllmann AJ, Association of *Borrelia garinii* and *B. valaisiana* with songbirds in Slovakia. Appl Environ Microbiol. 2003;69:2825–30. 10.1128/AEM.69.5.2825-2830.2003 12732554 PMC154513

[20] Poupon M-A, Lommano E, Humair PF, Douet V, Rais O, Schaad M, Prevalence of *Borrelia burgdorferi* sensu lato in ticks collected from migratory birds in Switzerland. Appl Environ Microbiol. 2006;72:976–9. 10.1128/AEM.72.1.976-979.2006 16391149 PMC1352204

[21] Giardina AR, Schmidt KA, Schauber EM, Ostfeld RS. Modeling the role of songbirds and rodents in the ecology of Lyme disease. Can J Zool. 2000;78:2184–97. 10.1139/z00-162

[22] Slowik TJ, Lane RS. Birds and their ticks in northwestern California: minimal contribution to *Borrelia burgdorferi* enzootiology. J Parasitol. 2001;87:755–61. 11534638 10.1645/0022-3395(2001)087[0755:BATTIN]2.0.CO;2

[23] LoGiudice K, Ostfeld RS, Schmidt KA, Keesing F. The ecology of infectious disease: effects of host diversity and community composition on Lyme disease risk. Proc Natl Acad Sci U S A. 2003;100:567–71. 10.1073/pnas.0233733100 12525705 PMC141036

[24] Barbour AG, Maupin GO, Teltow GJ, Carter CJ, Piesman J. Identification of an uncultivable *Borrelia* species in the hard tick *Amblyomma americanum*: possible agent of a Lyme disease-like illness. J Infect Dis. 1996;173:403–9. 10.1093/infdis/173.2.403 8568302

[25] Bunikis J, Garpmo U, Tsao J, Berglund J, Fish D, Barbour AG. Sequence typing reveals extensive strain diversity of the Lyme borreliosis agents *Borrelia burgdorferi* in North America and *Borrelia afzelii* in Europe. Microbiology. 2004;150:1741–55. 10.1099/mic.0.26944-0 15184561

[26] Bennet L. Erythema migrans in primary health care [Doctoral thesis]. Malmö (Sweden): Lund University; 2005.

[27] Sibley CG, Ahlquist JE. Phylogeny and classification of the birds of the world. New Haven (CT): Yale University Press; 1990.

[28] Magnarelli LA, Anderson JF, Fish D. Transovarial transmission of *Borrelia burgdorferi* in *Ixodes dammini* (Acari:Ixodidae). J Infect Dis. 1987;156:234–6. 10.1093/infdis/156.1.234 3598218

[29] Piesman J, Happ CM. The efficacy of co-feeding as a means of maintaining *Borrelia burgdorferi*: a North American model system. J Vector Ecol. 2001;26:216–20. 11813659

[30] Bunikis J, Tsao J, Garpmo U, Berglund J, Fish D, Barbour AG. Typing of *Borrelia* relapsing fever group strains. Emerg Infect Dis. 2004;10:1661–4. 15498172 10.3201/eid1009.040236 PMC3320305

[31] Mather TN, Telford SR III, MacLachlan AB, Spielman A. Incompetence of catbirds as reservoirs for the Lyme disease spirochete (*Borrelia burgdorferi*). J Parasitol. 1989;75:66–9. 10.2307/3282938 2918445

[32] Hedenström A, Alerstam T. Optimum fuel loads in migratory birds: distinguishing between time and energy minimization. J Theor Biol. 1997;189:227–34. 10.1006/jtbi.1997.0505 9441816

[33] Kurtenbach K, De Michelis S, Etti S, Schafer SM, Sewell HS, Brade V, Host association of *Borrelia burgdorferi* sensu lato- the key role of host complement. Trends Microbiol. 2002;10:74–9. 10.1016/S0966-842X(01)02298-3 11827808

[34] Lane RS, Loye JE. Lyme disease in California: interrelationship of *Ixodes pacificus* (Acari: Ixodidae), the western fence lizard (*Sceloporus occidentalis*), and *Borrelia burgdorferi.* J Med Entomol. 1989;26:272–8. 2769705 10.1093/jmedent/26.4.272

[35] Shaw DJ, Grenfell BT, Dobson AP. Patterns of macroparasite aggregation in wildlife host populations. Parasitology. 1998;117:597–610. 10.1017/S0031182098003448 9881385

[36] Randolph SE, Miklisova D, Lysy J, Rogers DJ, Labuda M. Incidence from coincidence: patterns of tick infestations on rodents facilitate transmission of tick-borne encephalitis virus. Parasitology. 1999;118:177–86. 10.1017/S0031182098003643 10028532

[37] Korenberg EI. Seasonal population dynamics of *Ixodes* ticks and tick-borne encephalitis virus. Exp Appl Acarol. 2000;24:665–81. 10.1023/A:1010798518261 11227825

[38] Eisen L, Eisen RJ, Lane RS. The roles of birds, lizards, and rodents as hosts for the western black-legged tick *Ixodes pacificus.* J Vector Ecol. 2004;29:295–308. 15709249

[39] Tälleklint L, Jaenson TG. Infestation of mammals by *Ixodes ricinus* ticks (Acari: Ixodidae) in south-central Sweden. Exp Appl Acarol. 1997;21:755–71. 10.1023/A:1018473122070 9423270

[40] BirdLife International. Birds in Europe: population estimates, trends and conservation status. Cambridge (UK): BirdLife International.; 2004.

