# Correction: Vol. 12 No. 7

**DOI:** 10.3201/eid1208.C31208

**Published:** 2006-08

**Keywords:** Correction Vol. 12 No. 7, Pär Comstedt, error, errata, correction

In "Migratory Passerine Birds as Reservoirs of Lyme Borreliosis in Europe," by Pär Comstedt et al., an error occurred in the second sentence of the first paragraph of Acknowledgments. The sentence should read "This is report no. 214 from the Ottenby Bird Observatory."

The corrected text appears in the online article at http://wwwnc.cdc.gov/eid/article/12/7/06-0127_article.htm.

We regret any confusion these errors may have caused.

